# Time Latency-Centric Signal Processing: A Perspective of Smart Manufacturing †

**DOI:** 10.3390/s21217336

**Published:** 2021-11-04

**Authors:** Sharifu Ura, Angkush Kumar Ghosh

**Affiliations:** 1 Division of Mechanical and Electrical Engineering, Kitami Institute of Technology, 165 Koen-cho, Kitami 090-8507, Japan; 2 Graduate School of Engineering, Kitami Institute of Technology, 165 Koen-cho, Kitami 090-8507, Japan; d1971308044@std.kitami-it.ac.jp

**Keywords:** sensor signals, smart manufacturing, time latency, low data acquisition, delay domain

## Abstract

Smart manufacturing employs embedded systems such as CNC machine tools, programable logic controllers, automated guided vehicles, robots, digital measuring instruments, cyber-physical systems, and digital twins. These systems collectively perform high-level cognitive tasks (monitoring, understanding, deciding, and adapting) by making sense of sensor signals. When sensor signals are exchanged through the abovementioned embedded systems, a phenomenon called time latency or delay occurs. As a result, the signal at its origin (e.g., machine tools) and signal received at the receiver end (e.g., digital twin) differ. The time and frequency domain-based conventional signal processing cannot adequately address the delay-centric issues. Instead, these issues can be addressed by the delay domain, as suggested in the literature. Based on this consideration, this study first processes arbitrary signals in time, frequency, and delay domains and elucidates the significance of delay domain over time and frequency domains. Afterward, real-life signals collected while machining different materials are analyzed using frequency and delay domains to reconfirm its (the delay domain’s) significance in real-life settings. In both cases, it is found that the delay domain is more informative and reliable than the time and frequency domains when the delay is unavoidable. Moreover, the delay domain can act as a signature of a machining situation, distinguishing it (the situation) from others. Therefore, computational arrangements enabling delay domain-based signal processing must be enacted to effectively functionalize the smart manufacturing-centric embedded systems.

## 1. Introduction

Nowadays, the fourth industrial revolution, popularly known as Industry 4.0 [1] or smart manufacturing [2], has been fostering profound transformations in the traditional manufacturing landscape. It converges information and communication technologies (ICT), internet-based infrastructures (e.g., IoT), and web-based technologies (e.g., Web 3.0/4.0 or Semantic Web) for expediting high-level cognitive tasks such as monitoring and decision-making. Consequently, monitoring machining processes (e.g., milling, grinding, turning, and alike) and deciding the right course of action have become a remarkable research topic. Monitoring a machining process generally involves two aspects, namely machine-condition and process-condition monitoring [3,4]. Here, machine-condition monitoring means monitoring the machine parts (e.g., gears and bearings). On the other hand, process-condition monitoring means monitoring the process-relevant components (e.g., cutting tool and workpiece surface). In both aspects, sensor signals (e.g., cutting force, torque, surface roughness, vibration, acoustic emission (AE), and alike) obtained from the machining environment play a key role.

**Figure 1:**
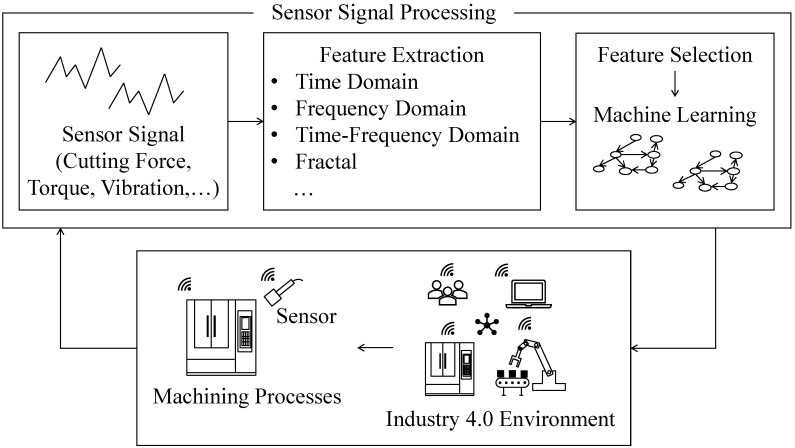
Context of sensor signal processing in Industry 4.0.

When a machining operation continues in a given smart manufacturing environment, as seen in Figure 1, sensors collect signals. The signals are generally processed in the time [5], frequency [6,7,8], and time-frequency [5,6,9] domains to extract the underlying features. In some cases, alternative processing methods, such as fractal-based [10], Approximate Entropy (ApEn)-based, and Sampling Entropy (SampEn)-based [11] methods, are used. Subsequently, the most relevant features are then selected using some computational arrangements (e.g., Principal Components Analysis (PCA) [12]). The abovementioned feature extraction and selection result in some machine learning algorithms. These algorithms become the core of an intelligent monitoring system.

However, the conventional signal processing methods (time, frequency, and time-frequency domain-based processing) require high data acquisition rates [13] and cannot mimic the dynamics underlying the signals [11]. Now, complex communication networks underlying smart manufacturing entail a phenomenon called time latency (also known as time delay) [14,15], which results in a low data acquisition rate. In addition, a high data acquisition rate requires a high data storage capacity and energy-intensive sensor networks [16]. Thus, a high data acquisition rate is not even desirable. As such, alternative methods are needed to tackle the abovementioned issues. Unfortunately, enough studies have not yet been conducted in this direction. This study fills this gap by adapting the delay domain-based signal processing method [17]. In particular, this study investigates the implications of the time latency domain or delay domain for understanding the dynamics underlying a given sensor signal. In addition, real-life sensor signals collected while machining metallic workpieces are analyzed by both conventional and delay domain-based approaches. Moreover, the efficacy of the delay domain in identifying different machining situations is also elucidated.

For better understanding, the rest of this article is organized as follows. Section 2 presents a literature review on sensor signal processing methods for machine- and process-condition monitoring. Section 3 presents the significances of the delay domain-based signal processing since the delay domain directly incorporates time latency associated with sensor signals. Section 4 presents a case study where delay domain-based signal processing is deployed to make sense of sensor signals of cutting force obtained from machining experiments. Section 5 presents the efficacy of delay domain-based processing where it is shown that the delay domain can distinguish different machining situations more effectively than the frequency domain. Finally, Section 6 provides the concluding remarks of this study.

## 2. Literature Review

Sensor signal processing is a mainspring for machine- and process-condition monitoring in manufacturing. Its role has been intensified due to the advent of Industry 4.0-centric embedded systems (cyber-physical systems and digital twins). Numerous researchers have been working on implementing the existing signal processing techniques or even developing new ones. For the sake of better understanding, some of the recent articles on signal processing are briefly described below.

First, consider the techniques used in sensor signal processing for manufacturing. Jáuregui et al. [6] presented a method for tool condition monitoring (TCM) in high-speed micro-milling. It incorporates frequency and time-frequency analysis of cutting force and vibration signals, acquired at a sampling frequency of 38,200 Hz and 89,100 Hz, respectively. Zhou et al. [18] introduced a sound signal-based TCM approach. The approach incorporates signal processing in a time-frequency domain called Wavelet Transform Modulus Maxima (WTMM). Chen et al. [5] proposed a method for monitoring chatter under different cutting conditions in milling. It incorporates time series analysis (using Recurrence Quantitative Analysis or RQA) of cutting force signals acquired at a sampling frequency of 8 kHz. Bi et al. [19] developed a method for monitoring grinding wheels while grinding brittle materials. The monitoring method acquires acoustic emission (AE) signals at a sampling frequency of 1 MHz and incorporates time and frequency analysis. Zhou and Xue [8] stressed the importance of multiple sensor signal analysis for developing intelligent monitoring systems. They also developed a multi-sensory monitoring system for TCM in milling. The system acquires cutting force, vibration, and AE signals at a sampling frequency of 50 kHz. Then, the signals are analyzed in the time, frequency, and time-frequency (Wavelet Packet Transform or WPT) domains. Li et al. [20] presented a multi-sensory system for identifying chatter in milling. It analyzes three signals, namely the cutting force, acceleration, and image ripple distance in the time and frequency domains (Wavelet Packet Decomposition or WPD). Segreto et al. [9] developed a multi-sensory system for estimating tool wear while turning hard-to-machine nickel-based alloys. The system acquires cutting force, AE, and vibration acceleration signals at a sampling frequency of 10 kHz, 10 kHz, and 3 kHz, respectfully. Then it (the system) analyzes these signals in the time-frequency (WPT) domain. Abubakr et al. [21] developed a model for detecting tool failure. The model acquires current, vibration, and AE signals at a sampling frequency of 250 Hz. Then it (the model) analyzes these signals in the time and frequency domains. Guo et al. [7] developed a system for predicting surface roughness due to grinding. The system analyzes the grinding force, vibration, and AE signals in the time and frequency domains. The sampling frequency for the force and vibration signals is 3200 Hz, and for the AE signals, it is 2000 kHz. Segreto et al. [22] presented a method for assessing surface roughness due to polishing. It analyzes the AE, strain, and current signals in time and time-frequency (WPT) domains. The corresponding sampling frequencies are 131 kHz, 16 kHz, and 0.1 kHz, respectively. Teti et al. [10] developed an artificial neural network (ANN)-based approach for monitoring tool health in drilling. It incorporates time, frequency, and fractal analysis of the thrust force and torque signals acquired at a sampling frequency of 10 kHz. Wang et al. [23] introduced an event-driven tool monitoring method for predicting the tools’ remaining useful life (RUL). It incorporates time and time-frequency analysis of cutting force signals to build the RUL prediction model. Jin et al. [24] introduced a method for monitoring bearing health. It acquires vibration signals at a sampling rate of 25.6 kHz. Then it incorporates time-frequency features underlying these signals for developing health index (HI) and predicting the bearing RUL. Bhuiyan et al. [25] investigated the relationship between tool wear and plastic deformation in turning. For this, AE signals are acquired at different sampling frequencies. The lowest sampling frequency studied was 50 kHz. The signals are analyzed in the frequency domain for the sake of investigation. Lee et al. [26] introduced a Kernel Principal Component Analysis (KPCA)-driven method for TCM in milling. It monitors tool wear using Kernel Density Estimation (KDE)-based T2-statistic and Q-statistic control charts. For this, current, AE, and vibration acceleration signals are acquired (from NASA’s milling dataset [27] at a minimum sampling frequency of 100 kHz) and analyzed.

In sum, the manufacturing-relevant sensor signals (e.g., cutting force, spindle load, AE, vibration, and alike) are commonly processed in the time, frequency, and time-frequency domains. Numerous authors have also studied the technical problems and limitations of the abovementioned sensor signal processing techniques. Some of the noteworthy studies are briefly described as follows.

Mahata et al. [28] articulated that time or frequency domain-based signal processing is ineffective for analyzing non-linear signals (e.g., signals underlying grinding wheel wear). They proposed a novel method defined as the Hilbert-Huang transform (HHT). The HHT acquires spindle power and vibration signals at a sampling frequency of 67 Hz and 10 kHz, respectively, and analyzes them. Espinosa et al. [11] articulated that traditional frequency analysis is not effective for unfolding the characteristics of non-linear signals, compared to the alternative analytics such as Approximate Entropy (ApEn) and Sampling Entropy (SampEn). Bayma et al. [29] proposed a Non-linear Output Frequency Response Functions (NOFRFs)-based approach for analyzing non-linear systems from the contexts of condition monitoring, fault diagnosis, and non-linear modal analysis. Bernard et al. [13] articulated that in the machine/process-condition monitoring research area, the methods used or introduced highly depend on high data acquisition rates. They emphasized the need for alternative methods that can perform with low data acquisition rates to meet some challenges of intelligent manufacturing, such as fast computation and low data storage. They also proposed a data-driven KDE function-based method for TCM. The method works with historical datasets of spindle load signals at a sampling frequency of 1/3 Hz. Cerna and Harvey [30] demonstrated that processing a signal dataset—associated with low sampling frequency—in the frequency domain results in a misleading representation due to aliasing. Du et al. [31] demonstrated that time latency and sampling rate must be synchronously optimized for improving control performance in a feedback control system. Lalouani et al. [16] described that acquiring data at higher sampling rates and its (data) transmission cause significant energy dissipation from a sensor system. They also articulated that suppressing energy dissipation is a prime requirement for the sustainable operation of a sensor system. They developed an energy optimization method, which deploys in-network processing to reduce the number of data transmissions. Halgamuge et al. [32] presented an overview of the sources of sensor power consumption. Some sources are: sensing signals at a sampling rate, signal conditioning, analog to digital conversion (ADC), reading/writing the sensed data in memory, and data transmitting. They also developed an energy model for estimating the life expectancy of wireless sensor networks (WSN). Wang and Chandrakasan [33] articulated that both the communication and computing energy need to be suppressed for prolonging the lifetimes of the wireless sensors in a multi-sensory network. For this, they stressed the need for an efficient signal processing method to extract meaningful information from the sensed data. McIntire et al. [34] described the requirements of Embedded Networked Sensor (ENS) systems from the viewpoint of critical environment monitoring. They articulated that the ENS must consume low energy. At the same time, the ENS must satisfy complex information processing to select proper sensor sampling. Marinkovic and Popovici [35] developed a method for suppressing the communication energy dissipation in a Wireless Body Area Network (WBAN) sensor node. The method adapts wireless wake-up functionality enabled by a Wake-Up Receiver (WUR). Brunelli et al. [36] articulated that computational tasks in a monitoring environment should consume less energy to guarantee an energy-efficient sensor network and data center.

In sum, conventional signal processing methods (time, frequency, and time-frequency domain-based methods) are inadequate for understanding the nature underlying non-linear and stochastic manufacturing signals. These methods highly depend on a higher data acquisition rate and complex computational arrangements, which create difficulties in storing the sensed data, consume more time to process the data, and cause energy dissipation from the sensor network. In addition, in reality, a low data acquisition rate is a possible outcome due to time latency (also known as delay) in complex communication networks underlying smart manufacturing systems [14,15,37]. Therefore, apart from the conventional methods, alternative methods must be investigated and incorporated for signal processing and handling the abovementioned difficulties from the smart manufacturing context. This study addresses this issue by adapting delay domain-based signal processing, as follows.

## 3. Significances of Time Latency and Delay Domain

As mentioned in the previous section, alternative signal processing methods are needed for unfolding the effect of time latency. For this, one straightforward way might be to adapt delay domain-based signal processing [17,38]. Few authors have embarked on this issue—delay domain-based signal processing and its implication in smart manufacturing. For example, Ullah [39] used delay domain-based processing for unfolding the dynamics underlying surface roughness signal datasets. Ullah and Harib [40] proposed a rule-based knowledge extraction process for simulating surface roughness where the rules are derived from the delay domain-based processing of historical roughness datasets. Wang and Li [41] used delay domain-based processing for the correlation analysis while developing a chaotic image encryption algorithm. Ghosh et al. [42] demonstrated the impact of time latency in signal processing. The authors articulated that the delay domain effectively encapsulates the dynamics underlying sensor signals when time latency is concerned. The authors also proposed a delay domain-driven hidden Markov process for developing sensor signal-based digital twins from the viewpoint of smart manufacturing [42,43]. However, more comprehensive studies are required to articulate the significances of time latency in sensor signals fully. For this, delay domain-based signal processing must be considered along with the conventional approaches (e.g., time and frequency domain-based signal processing).

**Figure 2:**
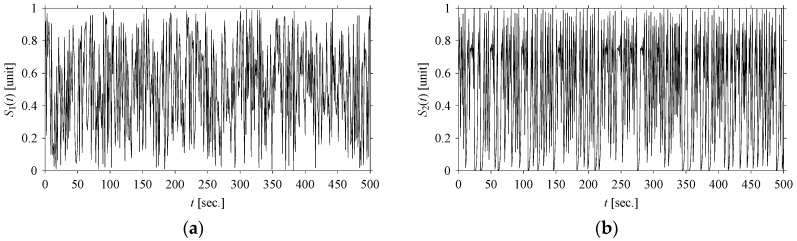
Time series of: (**a**) *S*_1_, (**b**) *S*_2_.

Delay domain-based signal processing means transferring a dataset (time series) to a space called the delay map [17,38]. The map considers a series of forward or backward values from the dataset based on a forward or backward delay parameter (a non-zero integer), respectively. For example, let {*A*(*t*) ∈ ℜ | *t* = 0, Δ*t*, 2Δ*t*, …} be a signal dataset, where ∆*t* is the sampling interval. Thus, (*t*,*A*(*t*)) is the corresponding time series. Now, let *d* be a non-zero integer to define the value of delay or time latency, i.e., *d* ∈ ℤ^+^. As such, the dataset of ordered pairs {(*A*(*t*),*A*(*t* ± *D*))|*D* = *d* × Δ*t*, *t* = 0, Δ*t*, 2Δ*t*, …} becomes the corresponding delay map. The time series can be represented by indexing its elements using a pointer, if preferred. In this case, *A*(*t*) is replaced by *A*(*i*) (=*A*(*t*)), where *t* = *i* × Δ*t* and *i* is the pointer, *i* = 0, 1, …. As such, (*A*(*i*),*A*(*i* + *d*)) becomes the corresponding delay map. For better understanding, consider the example in Figure 2.

**Figure 3:**
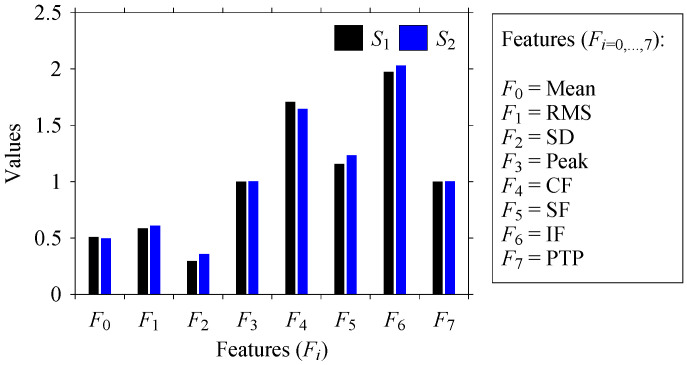
Quantifying *S*_1_ and *S*_2_ in the time domain.

Let *S*_1_ and *S*_2_ be the time series of two arbitrary signals, where the signal values are *S*_1_(*t*), *S*_2_(*t*) ∈ [0,1], *t* = 0, 1, …, as shown in Figure 2. Thus, the plots shown in Figure 2 are the time domain representations of the signals. Eight quantifiers (features) (*F*_0_, *F*_1_, *F*_2_, *F*_3_, *F*_4_, *F*_5_, *F*_6_, *F*_7_) = (Mean, Root Mean Square (RMS), Standard Deviation (SD), Peak, Crest Factor (CF), Shape Factor (SF), Impulse Factor (IF), and Peak to Peak (PTP)) are used to quantify *S*_1_ and *S*_2_ in the time domain. The values of the features are plotted in Figure 3 for both signals. As seen in Figure 3, the values of all features of *S*_1_ and *S*_2_ are almost the same. Therefore, as far as the time domain is concerned, *S*_1_ and *S*_2_ are two very similar signals.

**Figure 4:**
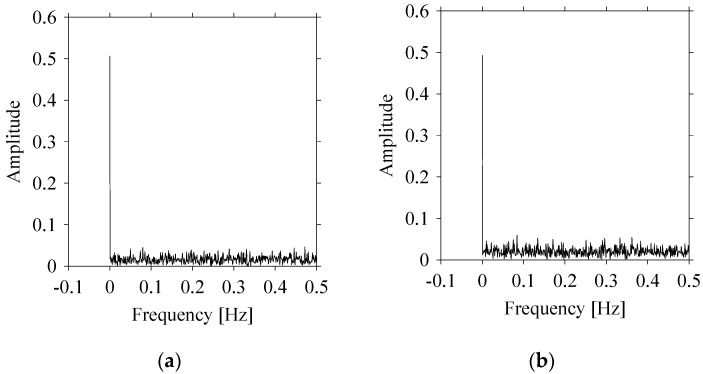
Quantifying *S*_1_ and *S*_2_ in the frequency domain. (**a**) The frequency domain of *S*_1_. (**b**) The frequency domain of *S*_2_.

Let us quantify the signals in the frequency domain. For this, the Fast Fourier Transform (FFT) is performed on the datasets shown in Figure 2 and the amplitude-frequency diagrams of *S*_1_ and *S*_2_ are constructed, as shown in Figure 4. As seen in Figure 4, the frequencies underlying *S*_1_ (Figure 4a) and *S*_2_ (Figure 4b) also exhibit a very similar pattern.

**Figure 5:**
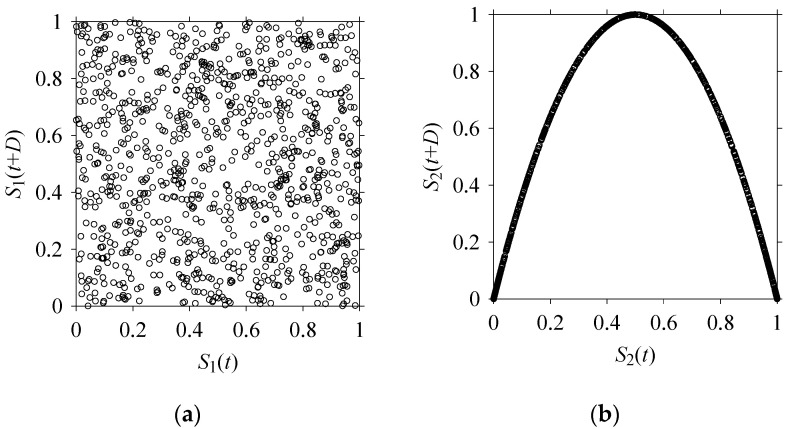
Delay maps (for *d* = 1) of: (**a**) *S*_1_, (**b**) *S*_2_.

Finally, consider delay domain-based analysis of *S*_1_ and *S*_2_. For this, two delay maps, as shown in Figure 5a,b for *S*_1_ and *S*_2_, respectively, are constructed. The *d* (delay parameter) is set to be 1. Consider the delay map *S*_1_ (Figure 5a). In this case, the points are randomly distributed on the delay map, revealing the fact that the value of signal at a point of time, *S*_1_(*t*), can change to a value taken from the interval [0,1] randomly in the next point of time, (*S*_1_(*t*+1)). On the other hand, consider the delay map shown in Figure 5b. A systematic pattern (the points are organized on parabolic curve) is shown in the delay map. This means that value of the signal at a point of time, *S*_2_(*t*), cannot change to a value taken from the interval [0,1] randomly in the next point of time, (*S*_2_(*t*+1)); it follows an order. This can also be described from the viewpoint of the entropy of information [44]. As reported in Appendix A, the entropy of information underlying the delay map of *S*_2_ (Figure 5b) is much less than that of *S*_1_ (Figure 5a), revealing the fact that *S*_2_ follows a very systematic pattern whereas *S*_1_ is random by nature. Note that the mathematical formulations for calculating the entropy of information are also described in detail in Appendix A.

Nevertheless, from the above time domain, frequency domain, and delay domain analyses, it is clear that delay domain-based analysis is more informative compared to time domain and frequency domain-based analyses for understanding the dynamics underlying *S*_1_ and *S*_2_. Thus, the delay domain is a powerful means to process signals.

## 4. Manufacturing Signal Processing

The previous section shows the significance of signal processing in the delay domain using two arbitrary signals. In this section, real-life signals are considered to see whether or not the delay domain remains significant in real-life settings. In particular, cutting force signals obtained by performing machining experiments according to the settings shown in Table 1 are considered.

**Figure 6:**
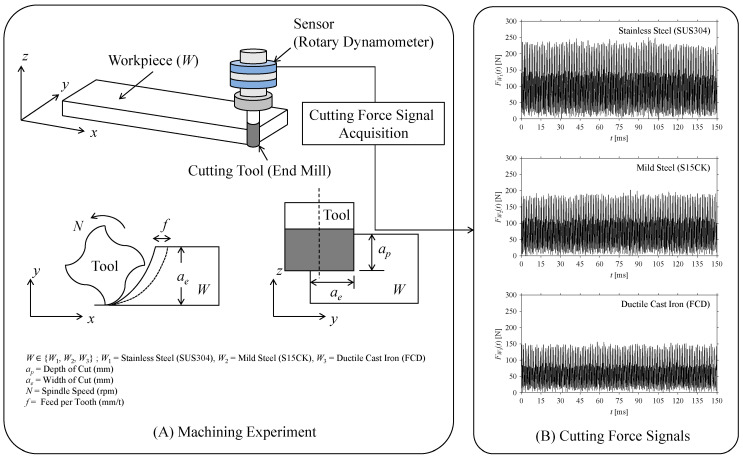
Outline of the machining experiment and signal acquisition.

Consider a machining experiment, where a set of workpiece specimens denoted as *W*, ∀*W* ∈ {*W*_1_, *W*_2_, *W*_3_} undergo end milling. Here, *W*_1_, *W*_2_, and *W*_3_ refer to the workpiece specimens made of stainless steel (JIS: SUS304), mild steel (JIS: S15CK), and ductile cast iron (JIS: FCD), respectively. Table 1 summarizes corresponding machining conditions (e.g., spindle speed *N* (rpm), feed per tooth *f* (mm/tooth), depth of cut *a_p_* (mm), width of cut *a_e_* (mm), and alike) in detail. Figure 6 schematically illustrates the outline of the experiment and relative conditions (see the segment denoted as A).

As seen in Figure 6, the corresponding cutting force signals are obtained using a sensor called rotary dynamometer (also described in Table 1) while machining *W*. Let, *F_W_*(*t*), *t* = 0, Δ*t*, 2Δ*t*, …, *m* × Δ*t*, be the force signals of *W*, as shown in the segment ‘B’ in Figure 6. Here, Δ*t* denotes a sampling period of 0.02 ms. The goal is to analyze the *F_W_* under low data acquisition scenarios for understanding the underlying dynamics. Note that a low data acquisition scenario can evolve from two different cases: (1) a short sampling window and (2) a low sampling rate due to time latency or delay. For this, the *F_W_* is analyzed considering both the abovementioned cases, as described in the following sub-sections, respectively.

### 4.1. Analyzing Sensor Signals under Different Sampling Windows

In Industry 4.0-centric systems, the sensor signal sampling windows may vary due to physical limitations and suppressing energy dissipation. Therefore, signals sampled using different sampling windows must be considered. Each piece of sampled signals can then be analyzed in the time, frequency, and delay domains, respectively.

**Figure 7:**
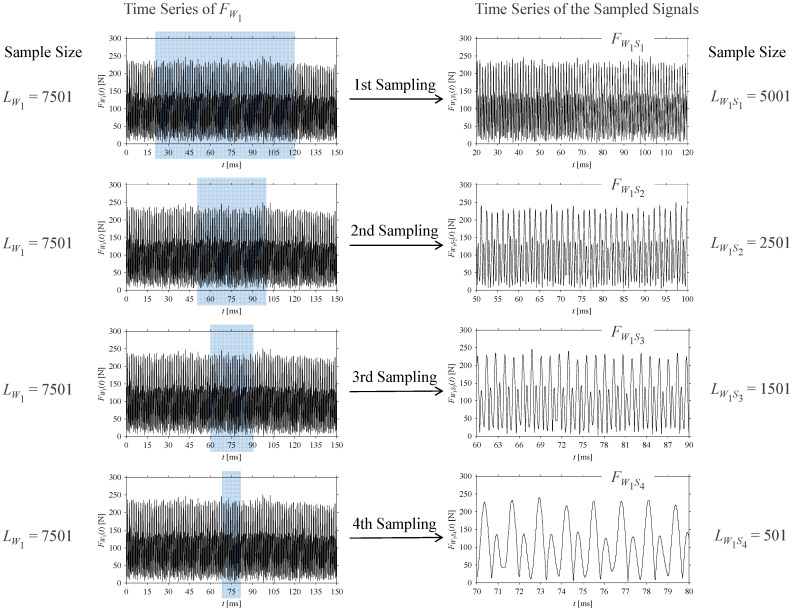
Sampling the cutting force signal for machining stainless steel (SUS304).

Consider the cutting force signal obtained while machining stainless steel (SUS304), i.e., *F*_*W*_1__. Figure 7 shows its time series. As seen in Figure 7, the sample size of *F*_*W*_1__ is denoted as *L*_*W*_1__, where *L*_*W*_1__ = 7501. *F*_*W*_1__ is sampled four times using four different sampling windows (light blue colored regions in the time series of *F*_*W*_1__). This results in four new signals denoted as *F*_*W*_1_*S*_1__, …, *F*_*W*_1_*S*_4__. The corresponding sample sizes are denoted as *L*_*W*_1_*S*_1__, …, *L*_*W*_1_*S*_4__, where *L*_*W*_1_*S*_1__ = 5001, *L*_*W*_1_*S*_2__ = 2501, *L*_*W*_1_*S*_3__ = 1501, and *L*_*W*_1_*S*_4__ = 501.

**Figure 8:**
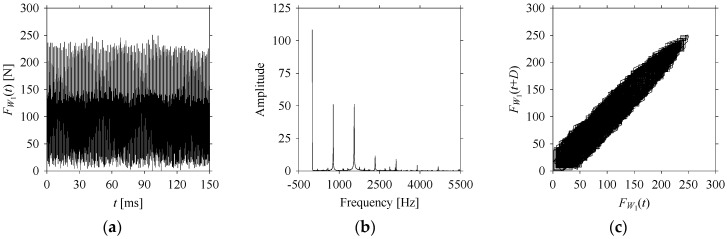
*F*_*W*_1__ in the form of: (**a**) time series, (**b**) fast Fourier Transformation (FFT), and (**c**) delay map (*d* = 1).

**Figure 9:**
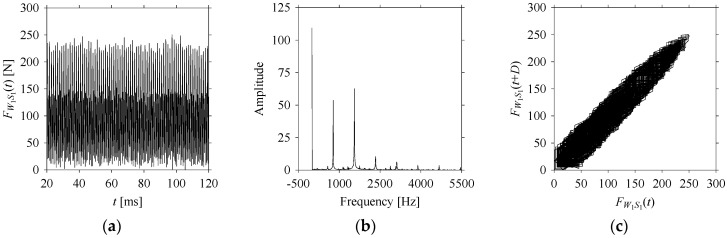
*F*_*W*_1_*S*_1__ in the form of: (**a**) time series, (**b**) fast Fourier Transformation (FFT), and (**c**) delay map (*d* = 1).

**Figure 10:**
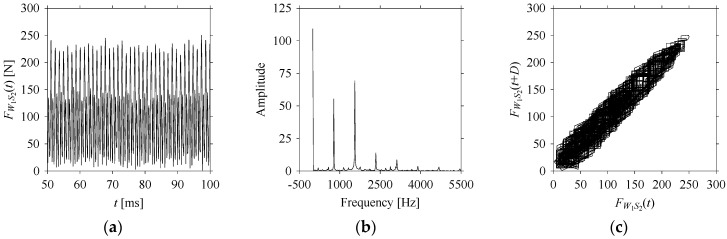
*F*_*W*_1_*S*_2__ in the form of: (**a**) time series, (**b**) fast Fourier Transformation (FFT), and (**c**) delay map (*d* = 1).

**Figure 11:**
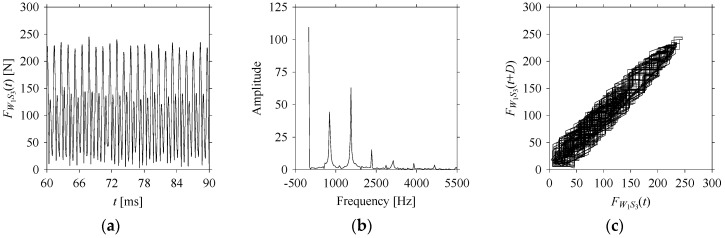
*F*_*W*_1_*S*_3__ in the form of: (**a**) time series, (**b**) fast Fourier Transformation (FFT), and (**c**) delay map (*d* = 1).

**Figure 12:**
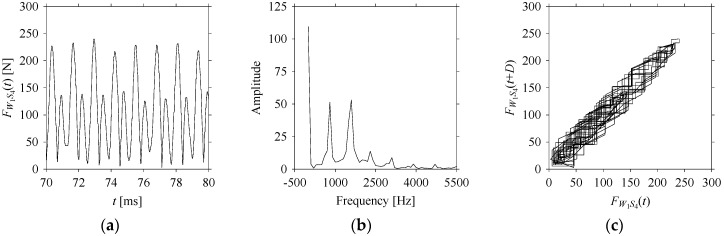
*F*_*W*_1_*S*_4__ in the form of: (**a**) time series, (**b**) fast Fourier Transformation (FFT), and (**c**) delay map (*d* = 1).

For the sake of analysis, the signals *F*_*W*_1__ and *F*_*W*_1_*S*_1__, …, *F*_*W*_1_*S*_4__ are transferred to the frequency domain (using FFT) and delay domain (using *d* = 1, as described in Section 3). As such, Figure 8 shows the time series, FFT, and delay map for *F*_*W*_1__. Figure 9, Figure 10, Figure 11 and Figure 12 show the time series, FFT, and delay map for *F*_*W*_1_*S*_1__, …, *F*_*W*_1_*S*_4__, respectively.

As seen in Figure 8b, the prominent frequencies underlying *F*_*W*_1__ are 0 Hz, 780 Hz, 1560 Hz, 2333.333 Hz, 3113.333 Hz, 3893.333 Hz, and 4673.333 Hz. As seen in Figure 9b, the prominent frequencies underlying *F*_*W*_1_*S*_1__ are 0 Hz, 780 Hz, 1560 Hz, 2340 Hz, 3110 Hz, 3890 Hz, and 4670 Hz. As seen in Figure 10b, the prominent frequencies underlying *F*_*W*_1_*S*_2__ are 0 Hz, 780 Hz, 1560 Hz, 2340 Hz, and 3120 Hz. As seen in Figure 11b, the prominent frequencies underlying *F*_*W*_1_*S*_3__ are 0 Hz, 766.6667 Hz, 1566.667 Hz, 2333.333 Hz, and 3133.333 Hz. As seen in Figure 12b, the prominent frequencies underlying *F*_*W*_1_*S*_4__ are 0 Hz, 800 Hz, 1600 Hz, 2300 Hz, and 3100 Hz. This means that the frequency information varies with the sampling window. In addition, the FFT pattern gets affected when the sampling window is shorter (see Figure 12b compared to Figure 8b).

On the other hand, the delay maps shown in Figure 8c, Figure 9c, Figure 10c, Figure 11c and Figure 12c exhibit similar characteristics under different sampling windows. In particular, the returns of points from one to another are identical. This means that the underlying nature of the *F*_*W*_1__ and *F*_*W*_1_*S*_1__, …, *F*_*W*_1_*S*_4__ are the same, regardless of the sample size. In addition, the density of points underlying the delay maps provides meaningful insight into the sample size of the signal. For example, the density of the delay map shown in Figure 12c is lighter compared to that of in Figure 8c, which mean the corresponding signal *F*_*W*_1_*S*_4__ (see Figure 12a) undergoes a low sampling window compared to the *F*_*W*_1__ (see Figure 8a).

Nevertheless, similar outcomes are observed for the other two workpiece specimens, i.e., *W*_2_ = mild steel (S15CK) and *W*_3_ = ductile cast iron (FCD), as described in Appendix B and Appendix C, respectively.

### 4.2. Analyzing Sensor Signals under Different Time Latencies 

Consider the cutting force signal obtained while machining stainless steel (SUS304), i.e., *F*_*W*_1__(*t*), *t* = 0, Δ*t*, 2Δ*t*, …, *m* × Δ*t* (which can also be seen in Figure 6). As mentioned before, here Δ*t* is the sampling period of 0.02 ms. To incorporate time latency or delay, Δ*t* is increased using the delay parameter *d* (non-zero integer), such as *d* × Δ*t*. For example, for *d* = 1, the sampling period remains 1 × 0.02 = 0.02 ms; for *d* = 2, the sampling period becomes 2 × 0.02 = 0.04 ms; and alike. As mentioned in Section 3, *d* × Δ*t* is simplified using *D*, where *D* = *d* × Δ*t*. As such, a set of time series is generated using *D* where *d* = 1, 2, …. The goal is to understand the dynamics underlying the cutting force. For this, the time series datasets are transferred to the frequency domains (using FFT) and corresponding delay domains. Table 2 shows the outcomes for some of the delays, i.e., *d* = 1, 5, 10, 20, 30, 40, 50, and 60.

As seen in Table 2, when *d* increases, the frequency information underlying the *F*_*W*_1__ gets affected. The prominent frequencies (see the FFT diagram for *d* = 1) are gradually lost due to aliasing [30] when *d* > 5 (see the FFT diagrams for *d* = 10, 20, 30, 40, 50, and 60). 

On the other hand, consider the delay maps consisting of the points (*F*_*W*_1__(*t*), *F*_*W*_1__(*t*+*D*)) shown in Table 2. When *d* = 1, the delay map exhibits a very systematic pattern. When *d* increases, the delay maps get more and more scattered (see the delay maps for *d* = 5, 10, 20, 30, 40, and 50). This means that the signal gets more and more chaotic due to the presence of delay. However, when *d* = 60, the corresponding delay map is somewhat systematic and similar to that of *d* = 5. This means that the underlying natures of these two signals are similar, regardless of the difference in the sampling rate. It is worth mentioning that the corresponding FFTs (see the FFTs for *d* = 5 and *d* = 60 shown in Table 2) are different and do not preserve the nature of the source signal. As such, delay domain-based representation is more informative for understanding the underlying nature of *F*_*W*_1__ under a low data acquisition rate due to time latency.

Nevertheless, similar outcomes are observed for the other two workpiece specimens, i.e., *W*_2_ = mild steel (S15CK) and *W*_3_ = ductile cast iron (FCD), as described in Appendix D and Appendix E, respectively.

To summarize, as seen in Table 3, real-life cutting force signals collected while machining different materials (stainless steel, mild steel, and ductile cast iron) are analyzed in the frequency and delay domains. This time, both the signal window and the amount of delay are varied to see their effect on signal processing. In the case of the signal window, it is found that both frequency and delay domains are effective for understanding the signals’ original nature for a larger window. On the other hand, the delay domain is more effective for smaller windows than the frequency domain. This is because frequency information gets lost or distorted when the sample size is smaller, whereas the delay domain retains the dynamics associated with the signals regardless of the sample size. In the case of varying delay, it is found that when delay increases, the frequency spectrum gets affected. The prominent frequencies of the original signal are gradually lost due to aliasing when the delay exceeds a critical value. On the other hand, when the delay increases, the delay domain gets more and more scattered. Furthermore, for some critical values of delay (one may be very high and the other may be very low), the delay domains exhibit similar characteristics, which is not the case for the frequency domains. Thus, when a very short window or low sampling rate (high delay) is used to analyze a signal, delay domains guarantee its (signal’s) original nature. This means that the delay domain-based representation is more robust in understating the nature of a sensor signal subjected to high time latency, a common phenomenon in Industry 4.0-relevant manufacturing environments.

## 5. Distinguishing Machining Situations Using Delay Maps

When machining is performed, the whole process undergoes different stages or situations. For example, at the onset and completion of machining, the cutting tool remains idle. Between the onset and completion, the tool machines the workpiece using a set of predefined cutting conditions. During cutting, the cutting conditions can be changed depending on the geometry or material of the workpiece. This creates the following situations: idle, idle to cutting, cutting with different cutting conditions/materials, cutting to idle, and idle. During this situation, an abnormality may happen (breakage of a tool, chatter vibration, and alike), adding other situations in the whole process. The Industry 4.0-centric systems must understand these situations on a real-time basis from sensor signals. Furthermore, the systems must coordinate among all machines under consideration and decide the right courses of action. Otherwise, the safety, economy, and quality of the other planning activities (e.g., maintenance scheduling) cannot be ensured [45]. Since delay maps effectively understand a signal’s hidden characteristics even though the signals are subjected to time latency, and the signal sampling window is a short one (as shown in Section 3 and Section 4), these maps can be employed to distinguish different machining situations. This section explores this possibility showing the efficacy of the delay domain. For the sake of better understanding, Section 5.1 describes the machining experiment and sensor signal acquisition. Afterward, Section 5.2 describes the pre-processing of the acquired signal. Finally, Section 5.3 describes the frequency and delay domain-based signal processing and the obtained results.

### 5.1. Sensor Signal Acquisition

**Figure 13:**
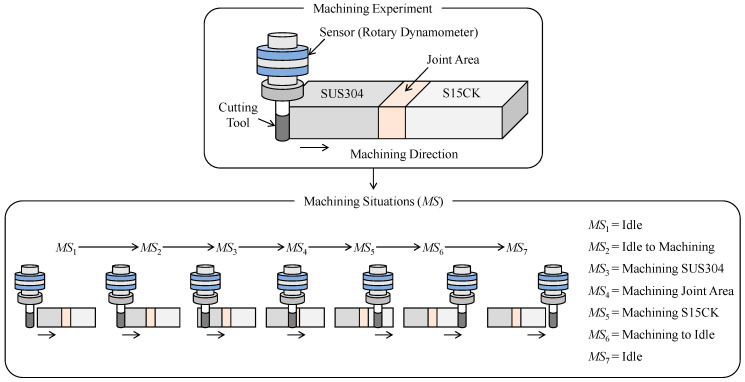
Machining situations underlying the machining experiment.

As seen in Figure 13, a multi-material workpiece made of stainless steel (JIS: SUS304) and mild steel (JIS: S15CK) is machined following an end milling process. The machining conditions are summarized in Table 4. The reasons for choosing a multi-material workpiece over a mono-material workpiece are as follows: (1) the usage of multi-material objects is increasing because these perform better in terms of material efficiency compared to their mono-material counterparts [44,46], and (2) Machining a multi-material object underlies more complex machining situations compared to machining a mono-material object.

However, the multi-material workpiece is machined from the hard-to-soft material direction (i.e., SUS304 to S15CK), as shown in Figure 13. As such, the machining involves a set of machining situations, denoted as *MS*, ∀*MS* ∈ {*MS*_1_, …, *MS*_7_}. Here, *MS*_1_ denotes the situation when the cutting tool is idle just before the machining. *MS*_2_ denotes the transition from an idle state to a machining state. *MS*_3_ denotes the machining of SUS304 segment. *MS*_4_ denotes the machining of the joint area (heat-affected area while joining SUS304 and S15CK). *MS*_5_ denotes the machining of S15CK segment. *MS*_6_ denotes the transition from a machining state to an idle state. Finally, *MS*_7_ denotes the situation when the tool is idle after completing the machining.

**Figure 14:**
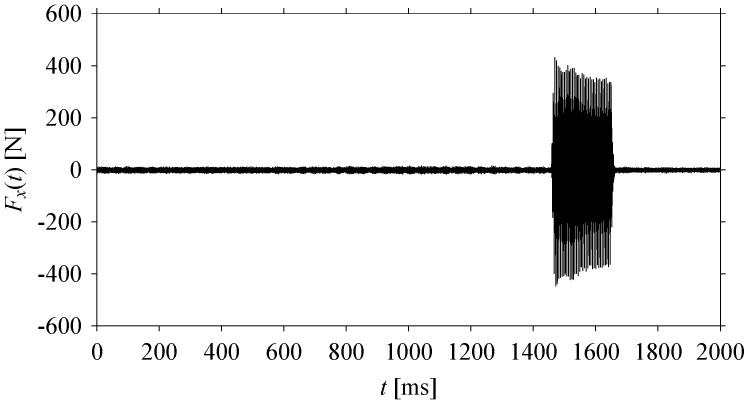
Time series of *F_x_*(*t*).

While the cutting tool is passed through the abovementioned situations, the corresponding machining force signals are recorded using a rotary dynamometer (as reported in Table 4). Let *F_o_* be the raw force signals, ∀*o* ∈ {*x*,*y*,*z*}. Here, *F_x_*, *F_y_*, and *F_z_* refer to the force signals along the *x*-axis, *y*-axis, and *z*-axis, respectively. The force signals in the *x*-axis, i.e., *F_x_*, are reported here. Figure 14 shows its time series, such as *F_x_*(*t*), *t* = 0, Δ*t*, 2Δ*t*, …. Here, Δ*t* is a sampling period of 0.02 ms.

### 5.2. Signal Pre-Processing

**Figure 15:**
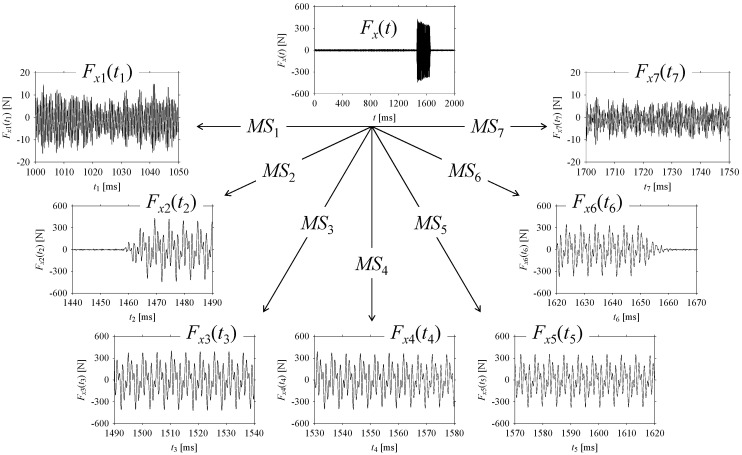
Sampling *F_x_*(*t*) based on machining situations (*MS*).

As seen in Figure 15, *F_x_*(*t*) is sampled to seven (7) fragments based on the abovementioned machining situations (*MS*). The fragments are denoted as *F_x_*_1_(*t*_1_), …, *F_x_*_7_(*t*_7_) corresponding to *MS*_1_, …, *MS*_7_, respectively. The sampling spans for *F_x_*_1_(*t*_1_), …, *F_x_*_7_(*t*_7_) are as follows: *t*_1_ = [*t*_start_,*t*_end_] = [1000,1050] ms, *t*_2_ = [*t*_start_,*t*_end_] = [1440,1490] ms, *t*_3_ = [*t*_start_,*t*_end_] = [1490,1540] ms, *t*_4_ = [*t*_start_,*t*_end_] = [1530,1580] ms, *t*_5_ = [*t*_start_,*t*_end_] = [1570,1620] ms, *t*_6_ = [*t*_start_,*t*_end_] = [1620,1670] ms, and *t*_7_ = [*t*_start_,*t*_end_] = [1700,1750] ms, respectively. Note that each fragment consists of 2501 samples.

Nevertheless, the sampled fragments (*F_x_*_1_(*t*_1_), …, *F_x_*_7_(*t*_7_)) are analyzed in the frequency and delay domains for distinguishing the corresponding machining situations (*MS*_1_, …, *MS*_7_).

### 5.3. Analysis in the Frequency and Delay Domains

**Figure 16:**
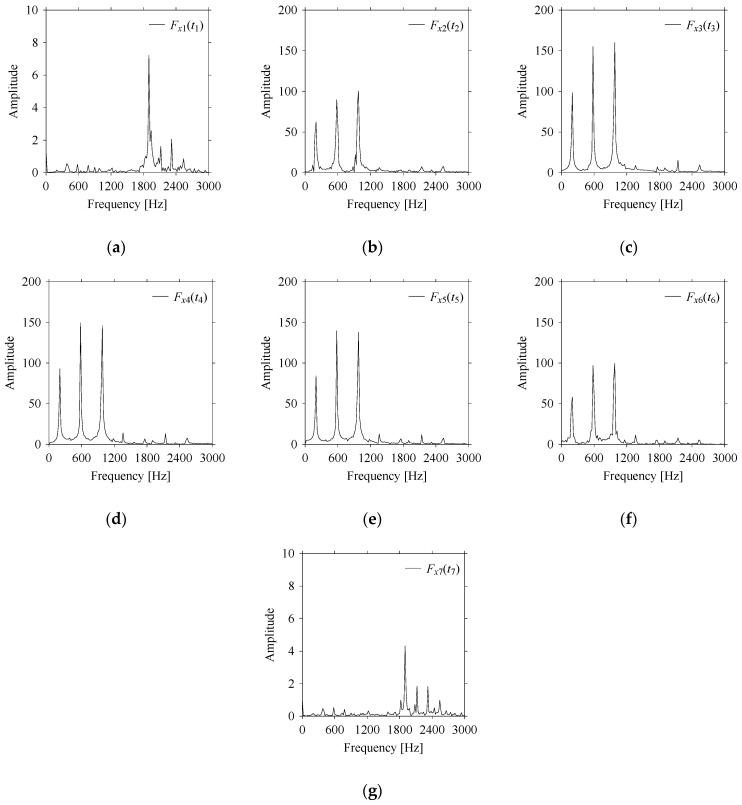
Frequency domain representations (FFTs) of the sampled signals: (**a**) FFT for *F_x_*_1_(*t*_1_), (**b**) FFT for *F_x_*_2_(*t*_2_), (**c**) FFT for *F_x_*_3_(*t*_3_), (**d**) FFT for *F_x_*_4_(*t*_4_), (**e**) FFT for *F_x_*_5_(*t*_5_), (**f**) FFT for *F_x_*_6_(*t*_6_), and (**g**) FFT for *F_x_*_7_(*t*_7_).

First, consider the frequency domain-based analysis. Figure 16a–g shows the frequency domain representations (FFT) of *F_x_*_1_(*t*_1_), …, *F_x_*_7_(*t*_7_) corresponds to *MS*_1_, …, *MS*_7_, respectively.

As seen in Figure 16a,g, the frequency components underlying *F_x_*_1_(*t*_1_) and *F_x_*_7_(*t*_7_) are the same, respectively. These frequency components are different from the frequency components underlying *F_x_*_2_(*t*_2_), …, *F_x_*_6_(*t*_6_), as shown in Figure 16b–f, respectively. In addition, the frequency components underlying *F_x_*_2_(*t*_2_), …, *F_x_*_6_(*t*_6_) are the same (see Figure 16b–f, respectively). This means that the frequency domain-based analysis is effective only for distinguishing the situations *MS*_1_ (underlying *F_x_*_1_(*t*_1_)) and *MS*_7_ (underlying *F_x_*_7_(*t*_7_)) from others (*MS*_2_, …, *MS*_6_ underlying *F_x_*_2_(*t*_2_), …, *F_x_*_6_(*t*_6_), respectively). It is not effective for distinguishing the situations *MS*_2_, …, *MS*_6_ (underlying *F_x_*_2_(*t*_2_), …, *F_x_*_6_(*t*_6_), respectively) from each other.

Now, consider the delay domain-based analysis. For this, a set of delay maps are constructed for each signals *F_x_*_1_(*t*_1_), …, *F_x_*_7_(*t*_7_) by varying the delay parameter *d* = 1, …, 100. Note that the delay maps are generated following the methodology described in Section 3 (which can also be seen in Section 4.2). Table 5 shows some of the delay maps corresponding to *d* = 1, 2, 5, 50, and 100.

As seen in Table 5, the delay maps of *F_x_*_1_(*t*_1_), …, *F_x_*_7_(*t*_7_) exhibit similar patterns when *d* is very small (*d* = 1 and 2). The maps become more and more chaotic with the increase in *d*, as observed in Table 5. For some specific delay parameter values, a delay map corresponding to a given machining situation exhibits a distinct pattern, although it is not usually the case. Nevertheless, the specific values of delay for which each delay map exhibits a distinct pattern are valuable for Industry 4.0-centric systems. In this case, the systems make a one-to-one correspondence between a given machining situation and its delay map, which is ideal for pattern recognition. This means that a delay map can be a signature of a machining situation. For example, for the signals reported in Figure 15, the delay maps exhibit unique patterns when *d* = 43, as shown in Table 6. In other words, the delay maps corresponding to *d* = 43 are the signatures of the machining situations *MS*_1_, …, *MS*_7_.

## 6. Concluding Remarks

This study elucidates the implications of time latency or delay in signal processing from the perspective of smart manufacturing. In smart manufacturing, signal processing frameworks mostly follow time and frequency analyses. In addition, there is no systematic study from the viewpoint of manufacturing practices where delay-related issues are addressed adequately. This study fills this gap by analyzing manufacturing-centric sensor signals (cutting force signals) in the delay domain in addition to time and frequency domains.

This study first studies two arbitrary chaotic signals that look alike in the time and frequency domains. However, their differences could not be understood delay until they are represented in the delay domain. This means that the delay domain must be incorporated to make sense of chaotic sensor signals. Chaotic sensor signals are common in smart manufacturing.

In the next step, real-life sensor signals relevant to manufacturing, particularly cutting force signals collected while machining different materials (stainless steel, mild steel, and ductile cast iron), are analyzed in frequency and delay domains. This time, both signal window and the amount of delay are varied to see their effect on signal processing. It is found that when delay increases, the frequency spectrum gets affected. The prominent frequencies of the original signal are gradually lost due to aliasing when the delay exceeds a critical value. On the other hand, when the delay increases, the delay domain gets more and more scattered. For some critical values of delay (one may be very high and the other may be very low), the delay domains exhibit similar characteristics, which is not the case for the frequency domains. Thus, when a very short window or low sampling rate (high delay) is unavoidable, the delay domains guarantee its (signal’s) original nature. This means that the delay domain-based signal processing is more robust in understating the nature of a sensor signal subjected to high time latency.

Lastly, the potential of the delay domain becoming a signature of a machining situation is studied using real-life signals. For this, sensor signals are sampled exhibiting seven different machining situations. The situations represent the onset of machining, completion of machining, machining of different materials, and transitions situations. For some specific delays, the delay maps make one-to-one correspondence with machining situations. This means that a delay domain of a machine situation is different from the delay domains of other machining situations for a critical delay. Consequently, a delay domain can be used as a signature of a machining situation.

In synopsis, computational arrangements enabling delay domain-based signal processing must accompany the smart manufacturing-centric embedded systems because such systems (CNC machine tools, PLC, robots, digital measuring instruments, cyber-physical systems, and digital twins) are subjected to acute time latency. 

Thus, the outcomes of this study contribute to the advancement of smart manufacturing.

## Figures and Tables

**Table 1:** Conditions for machining experiment.

Item	Description
Machine tool	Vertical machining center
	Make: Mori Seiki
	Model: NV5000
Cutting tool	Carbide Φ6 solid end mil
	Make: Mitsubishi Hitachi
	Model: EPP4060-P-CS
	Number of teeth (*n_t_*): 4
Sensor	Rotary dynamometer
	Make: Kistler
	Type: 9170A
Workpiece material	Stainless steel (JIS: SUS304)
	Mild steel (JIS: S15CK)
	Ductile cast iron (JIS: FCD)
Cutting velocity (*v_c_*)	220 m/min
Spindle speed (*N*)	11677 rpm
Feed per tooth (*f*)	0.1 mm/tooth
Feed rate (*v_f_*)	4671 mm/min
Depth of cut (*a_p_*)	1.0 mm
Width of cut (*a_e_*)	0.5 mm

**Table 2:** *F*_*W*_1__ in the form of time series, FFT, and delay map under varying delay (*d*).

Time Series	FFT	Delay Map
*d* = 1		
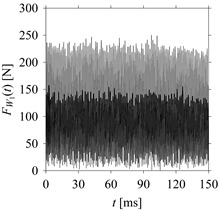	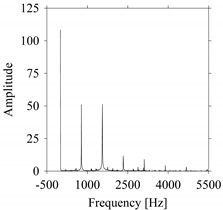	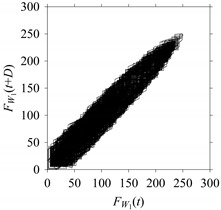
*d* = 5		
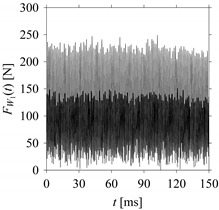	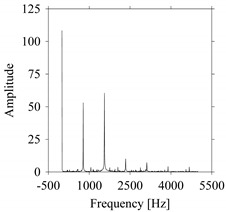	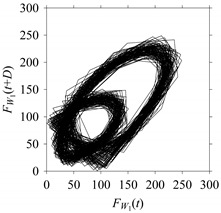
*d* = 10		
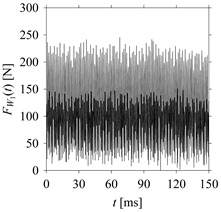	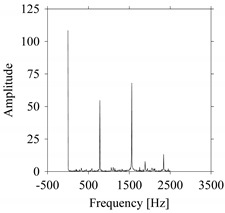	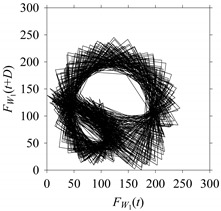
*d* = 20		
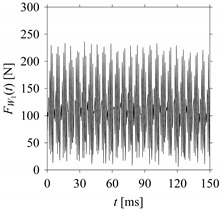	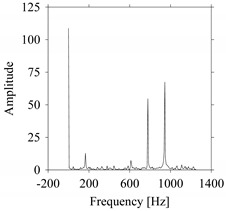	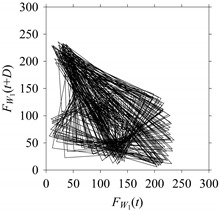
*d* = 30		
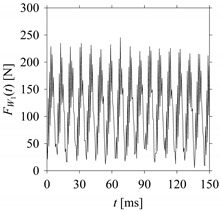	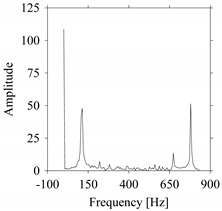	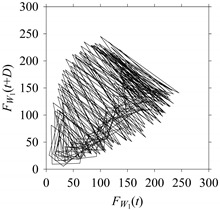
*d* = 40		
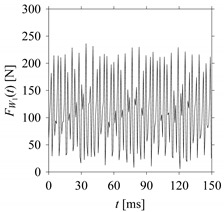	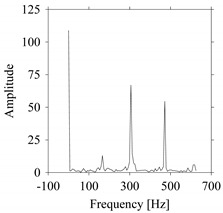	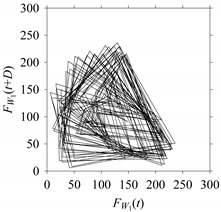
*d* = 50		
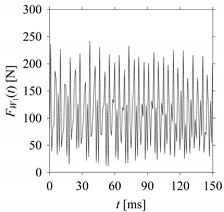	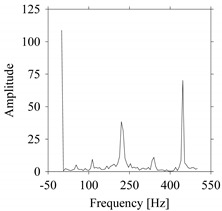	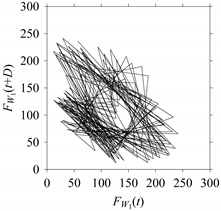
*d* = 60		
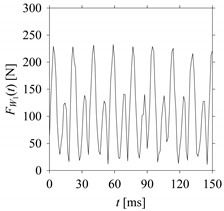	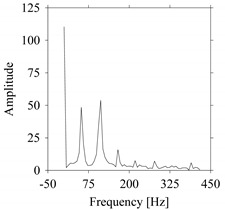	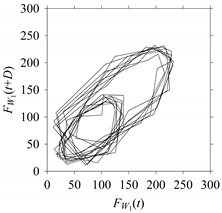

**Table 3:** Summary of observations from manufacturing signal processing.

**Analyzing Signals under Different Sampling Windows**		
**Sample Size**	**Observations**	
	**Frequency Domain**	**Delay Domain**
Larger	# Meaningful frequency information is prominent. # Effective for understanding the signals’ original nature.	# Point clouds’ density is higher. # The density clearly signifies the sample size. # Effective for understanding the signals’ original nature.
Smaller	# Frequency information significantly varies with the sample size. # Frequency information gets lost or distorted. # For smaller sample size, the fast Fourier transformation (FFT) exhibits a different pattern than that of the signal with larger sample size. # Not so effective for understanding the original nature underlying a signal.	# Point clouds’ density is lower. # The density clearly signifies the sample size. # Regardless of the sample size, the delay maps retain the dynamics. # Effective for understanding the original nature underlying a signal.
**Analyzing Signals under Different Time Latencies or Delay**		
**Delay,** ***d* = 1, …, 100**	**Observations**	
	**Frequency Domain**	**Delay Domain**
*d* < 5	# Meaningful frequency information is prominent. # Effective for understanding the signals’ original nature.	# Delay maps exhibit very systematic patterns. # Effective for understanding the signals’ original nature.
*d* = 5	# Meaningful frequency information is somewhat prominent. # Effective for understanding the signals’ original nature.	# Delay map exhibit somewhat systematic pattern. # Effective for understanding the signals’ original nature. # Delay map starts getting scattered.
*d* > 5	# Meaningful frequency information is gradually lost due to aliasing. # Not any more effective for understanding the signals’ original nature.	# Delay maps get scattered. # The signal starts to get chaotic. # The chaotic behavior of the delay map signifies the presence of the delay.
*d* = 60	# Same as above	# Delay map exhibits somewhat systematic pattern again (similar to the pattern for *d* = 5). # This phenomenon signifies that delay domain guarantees signals’ original nature for some critical values of delay (e.g., here 5 and 60). # This also signifies that delay domain is more robust in understanding the nature of a signal subjected to high time latency (or delay).
60 < *d* < 100	# Same as above	# Delay maps get scattered again.

**Table 4:** Machining conditions for machining a multi-material workpiece.

Item	Description
Machine tool	Vertical machining center
	Make: Mori Seiki
	Model: NV5000
Cutting tool	Carbide Φ6 solid end mil
	Make: Mitsubishi Hitachi
	Model: EPP4060-P-CS
	Number of teeth (*n_t_*): 4
Sensor	Rotary dynamometer
	Make: Kistler
	Type: 9170A
Workpiece material	Stainless steel (JIS: SUS304)—Mild steel (JIS: S15CK)
Cutting velocity (*v_c_*)	220 m/min
Spindle speed (*N*)	11,677 rpm
Feed per tooth (*f*)	0.2 mm/tooth
Feed rate (*v_f_*)	9341 mm/min
Depth of cut (*a_p_*)	2.0 mm
Width of cut (*a_e_*)	0.5 mm
Machining direction	SUS304 to S15CK

**Table 5:** Delay maps for *F_x_*_1_(*t*_1_), …, *F_x_*_7_(*t*_7_) (corresponds to *MS*_1_, …, *MS*_7_) when *d* = 1, 2, 5, 50, and 100.

**Machining Situations (*MS*)**						
*MS*_1_	*MS*_2_	*MS*_3_	*MS*_4_	*MS*_5_	*MS*_6_	*MS*_7_
**Sampled Signals Corresponding to *MS***						
*F_x_*_1_(*t*_1_)	*F_x_*_2_(*t*_2_)	*F_x_*_3_(*t*_3_)	*F_x_*_4_(*t*_4_)	*F_x_*_5_(*t*_5_)	*F_x_*_6_(*t*_6_)	*F_x_*_7_(*t*_7_)
**Delay Maps for Varying *d***						
*d* = 1						
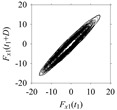	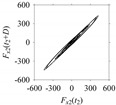	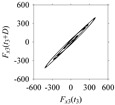	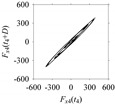	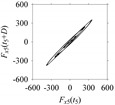	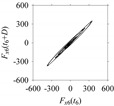	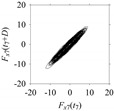
*d* = 2						
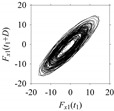	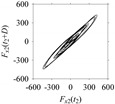	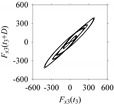	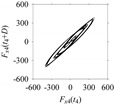	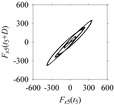	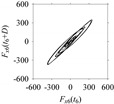	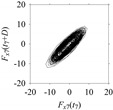
*d* = 5						
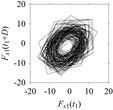	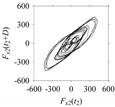	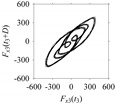	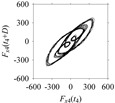	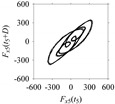	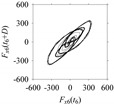	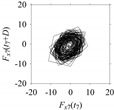
*d* = 50						
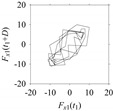	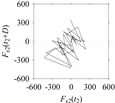	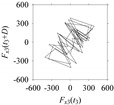	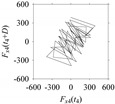	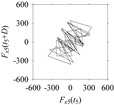	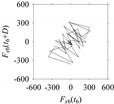	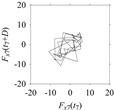
*d* = 100						
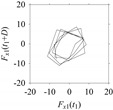	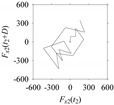	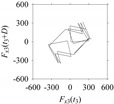	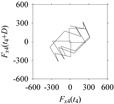	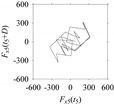	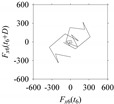	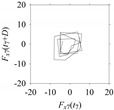

**Table 6:** Delay maps for *F_x_*_1_(*t*_1_), …, *F_x_*_7_(*t*_7_) (corresponds to *MS*_1_, …, *MS*_7_) when *d* = 43.

**Machining Situations (*MS*)**						
*MS*_1_	*MS*_2_	*MS*_3_	*MS*_4_	*MS*_5_	*MS*_6_	*MS*_7_
**Sampled Signals Corresponding to *MS***						
*F_x_*_1_(*t*_1_)	*F_x_*_2_(*t*_2_)	*F_x_*_3_(*t*_3_)	*F_x_*_4_(*t*_4_)	*F_x_*_5_(*t*_5_)	*F_x_*_6_(*t*_6_)	*F_x_*_7_(*t*_7_)
**Delay Maps for *d* = 43**						
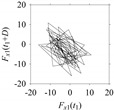	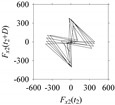	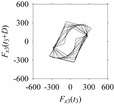	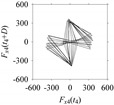	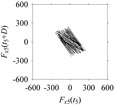	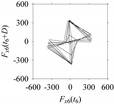	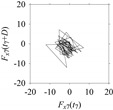

**Figure A1:**
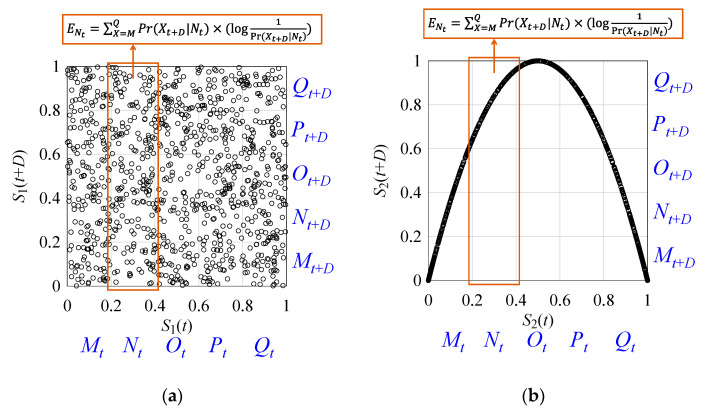
The concept of calculating entropy of information underlying the delay maps of: (**a**) *S*_1_ and (**b**) *S*_2_.

**Figure A2:**
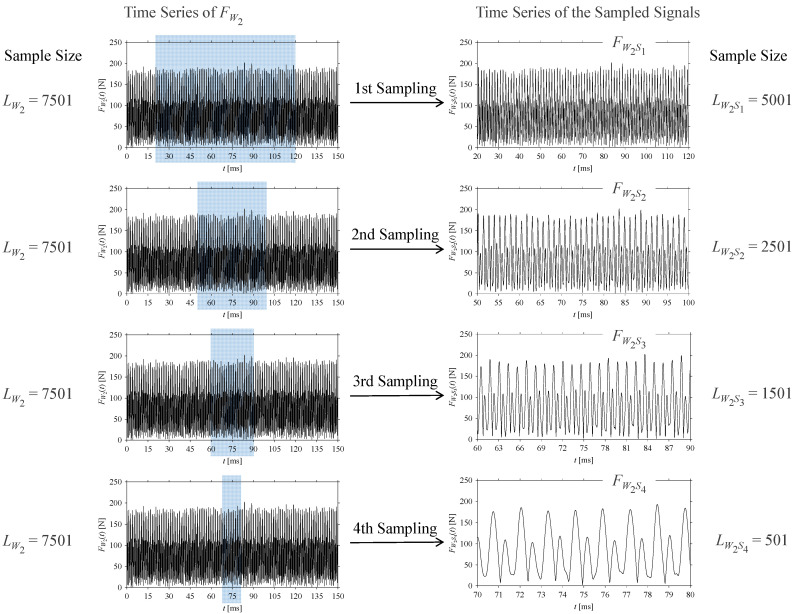
Sampling the cutting force signal for machining mild steel (S15CK).

**Figure A3:**
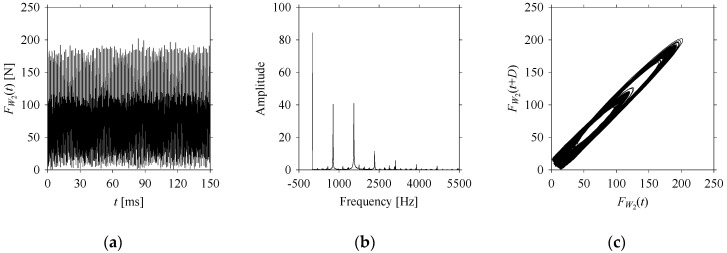
*F*_*W*_2__ in the form of: (**a**) time series, (**b**) fast Fourier Transformation (FFT), and (**c**) delay map (*d* = 1).

**Figure A4:**
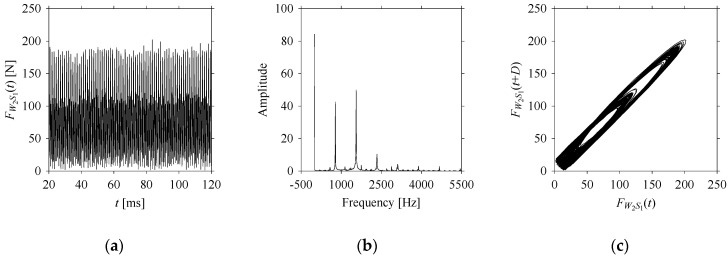
*F*_*W*_2_*S*_1__ in the form of: (**a**) time series, (**b**) fast Fourier Transformation (FFT), and (**c**) delay map (*d* = 1).

**Figure A5:**
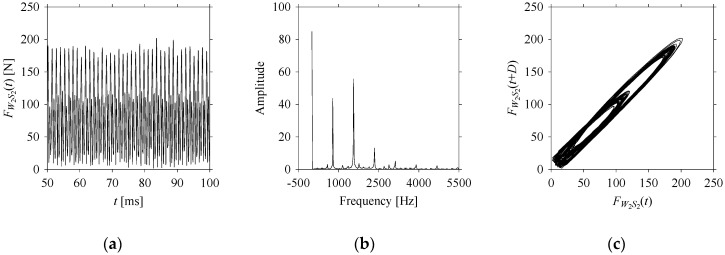
*F*_*W*_2_*S*_2__ in the form of: (**a**) time series, (**b**) fast Fourier Transformation (FFT), and (**c**) delay map (*d* = 1).

**Figure A6:**
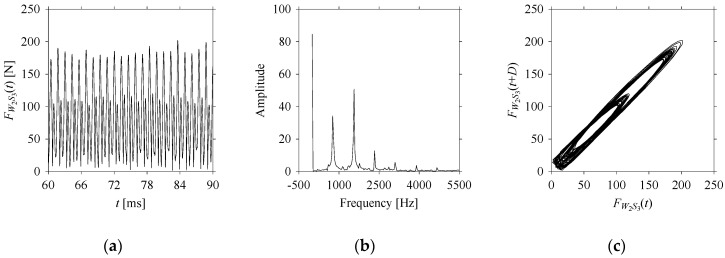
*F*_*W*_2_*S*_3__ in the form of: (**a**) time series, (**b**) fast Fourier Transformation (FFT), and (**c**) delay map (*d* = 1).

**Figure A7:**
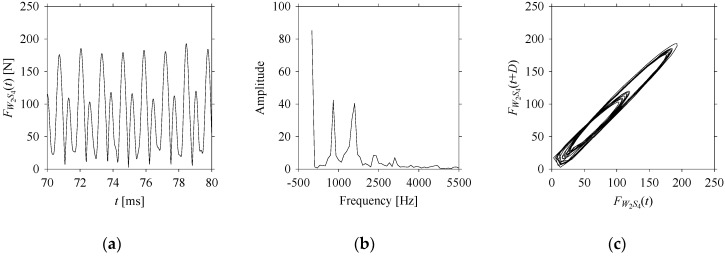
*F*_*W*_2_*S*_4__ in the form of: (**a**) time series, (**b**) fast Fourier Transformation (FFT), and (**c**) delay map (*d* = 1).

**Figure A8:**
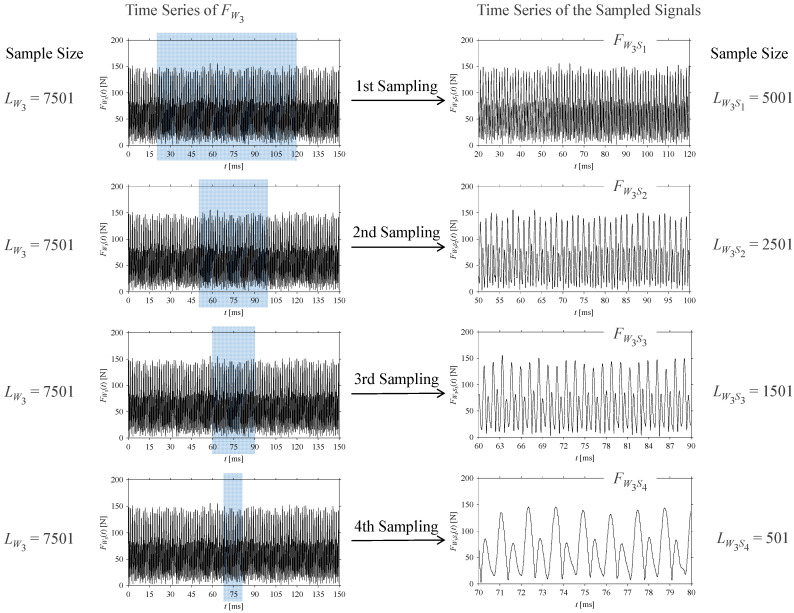
Sampling the cutting force signal for machining ductile cast iron (FCD).

**Figure A9:**
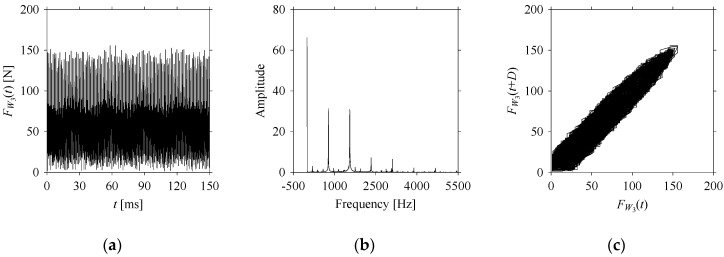
*F*_*W*_3__ in the form of: (**a**) time series, (**b**) fast Fourier Transformation (FFT), and (**c**) delay map (*d* = 1).

**Figure A10:**
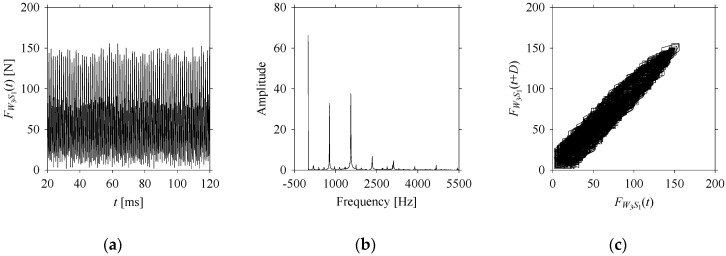
*F*_*W*_3_*S*_1__ in the form of: (**a**) time series, (**b**) fast Fourier Transformation (FFT), and (**c**) delay map (*d* = 1).

**Figure A11:**
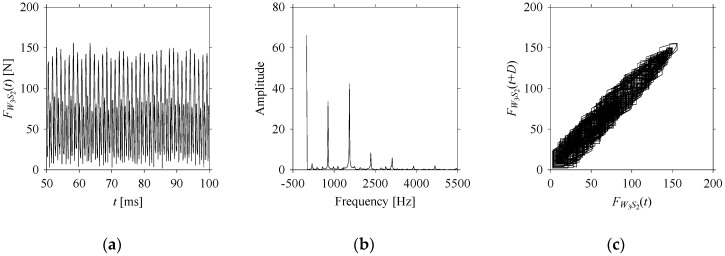
*F*_*W*_3_*S*_2__ in the form of: (**a**) time series, (**b**) fast Fourier Transformation (FFT), and (**c**) delay map (*d* = 1).

**Figure A12:**
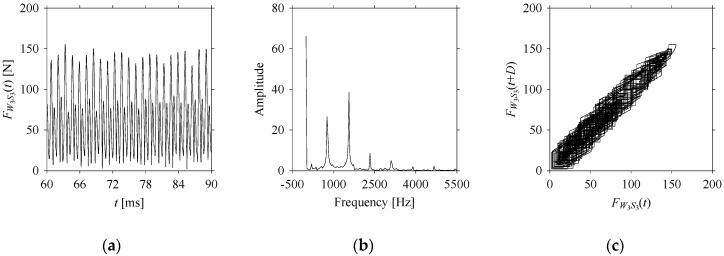
*F*_*W*_3_*S*_3__ in the form of: (**a**) time series, (**b**) fast Fourier Transformation (FFT), and (**c**) delay map (*d* = 1).

**Figure A13:**
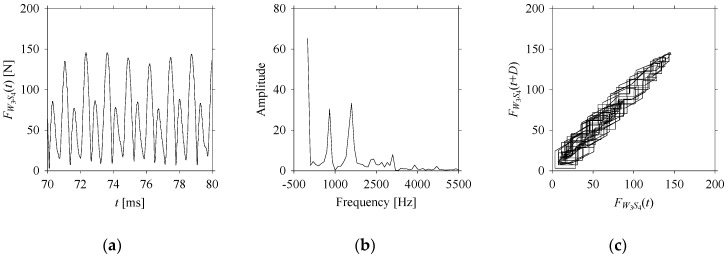
*F*_*W*_3_*S*_4__ in the form of: (**a**) time series, (**b**) fast Fourier Transformation (FFT), and (**c**) delay map (*d* = 1).

## Data Availability

The data are available from the corresponding author upon request.

## Appendix A. Entropy of Information

First, consider the delay maps of *S*_1_ and *S*_2_ (see Figure 5a,b).

Since {*S*_1_,*S*_2_} ⊇ [0,1], let *M*, *N*, *O*, *P*, *Q* be five mutually exclusive intervals in [0,1] so that *M* < *N* < *O* < *P* < *Q* and *M* ∪ *N* ∪ *O* ∪ *P* ∪ *Q* = [0,1]. The most straightforward definitions of the intervals are as follows: *M* = [0,0.2), *N* = [0.2,0.4), *O* = [0.4,0.6), *P* = [0.6,0.8), and *Q* = [0.8,1] as shown in Figure A1.

The abovementioned intervals are the states of {*S*_1_,*S*_2_}. This means that if {*S*_1_,*S*_2_} ∈ *M*, then the state of {*S*_1_,*S*_2_} is *M*. If {*S*_1_,*S*_2_} ∈ *N*, then the state of {*S*_1_,*S*_2_} is *N*. If {*S*_1_,*S*_2_} ∈ *O*, then the state of {*S*_1_,*S*_2_} is *O*. If {*S*_1_,*S*_2_} ∈ *P*, then the state of {*S*_1_,*S*_2_} is *P*. Finally, if {*S*_1_,*S*_2_} ∈ *Q*, then the state of {*S*_1_,*S*_2_} is *Q*.

Now, the dynamics underlying {*S*_1_,*S*_2_} can be represented by the probabilities of the transitions from all possible states. These transition probabilities can be denoted as *Pr*(*X_t_*_+*D*_ | *Y_t_*) where ∀*X_t_*_+*D*_ ∈{*M_t_*_+*D*_, *N_t_*_+*D*_, *O_t_*_+*D*_, *P_t_*_+*D*_, *Q_t_*_+*D*_} ∈ [0,1], ∀*Y_t_* ∈ {*M_t_*, *N_t_*, *O_t_*, *P_t_*, *Q_t_*}, *M_t_*_+*D*_ = *M_t_* = *M*, *N_t_*_+*D*_ = *N_t_* = *N*, *O_t_*_+*D*_ = *O_t_* = *O*, *P_t_*_+*D*_ = *P_t_* = *P*, and *Q_t_*_+*D*_ = *Q_t_* = *Q*, as shown in Figure A1.

The abovementioned transition probabilities create an entity defined as the Transition Probability (*Pr*) Matrix (*TM*) as follows.

(A1)
$$TM=\left[\begin{array}{ccc} Pr\left(M_{t+D}|M_{t}\right) & \cdots & Pr\left(Q_{t+D}|M_{t}\right)\\ \vdots & \ddots & \vdots \\ Pr\left(M_{t+D}|Q_{t}\right) & \cdots & Pr\left(Q_{t+D}|Q_{t}\right) \end{array}\right].\;$$

From the abovementioned *TM*, the entropy of information, denoted as *E_Yt_*, *Y_t_* ∈ {*M_t_*, *N_t_*, *O_t_*, *P_t_*, *Q_t_*}, is calculated as follows. This scenario is also schematically illustrated in Figure A1.

(A2)
$$E_{M_{t}}=\overset{Q}{\underset{X=M}{\sum }}Pr\left(X_{t+D}|M_{t}\right)\times \left(\log\frac{1}{Pr\left(X_{t+D}|M_{t}\right)}\right)$$

(A3)
$$E_{N_{t}}=\overset{Q}{\underset{X=M}{\sum }}Pr\left(X_{t+D}|N_{t}\right)\times \left(\log\frac{1}{Pr\left(X_{t+D}|N_{t}\right)}\right)$$

(A4)
$$E_{O_{t}}=\overset{Q}{\underset{X=M}{\sum }}Pr(X_{t+D}|O_{t})\times \left(\log\frac{1}{Pr\left(X_{t+D}|O_{t}\right)}\right)$$

(A5)
$$E_{P_{t}}=\overset{Q}{\underset{X=M}{\sum }}Pr(X_{t+D}|P_{t})\times \left(\log\frac{1}{Pr\left(X_{t+D}|P_{t}\right)}\right)$$

(A6)
$$E_{Q_{t}}=\overset{Q}{\underset{X=M}{\sum }}Pr(X_{t+D}|Q_{t})\times \left(\log\frac{1}{Pr\left(X_{t+D}|Q_{t}\right)}\right)$$

The abovementioned *E_Yt_*, *Y_t_* ∈ {*M_t_*, *N_t_*, *O_t_*, *P_t_*, *Q_t_*}, creates an Entropy Matrix (*EIM*), as follows,

(A7)
$$EIM=\left[\begin{array}{c} E_{M_{t}}\\ \begin{array}{c} E_{N_{t}}\\ \begin{array}{c} E_{O_{t}}\\ \begin{array}{c} E_{P_{t}}\\ E_{Q_{t}} \end{array} \end{array} \end{array} \end{array}\right].$$

Therefore, following the abovementioned mathematical formulations, *EIM* for *S*_1_ and *S*_2_ have been calculated, as shown in Equations (A8) and (A9), respectively.

(A8)
$$EIM_{S_{1}}=\left[\begin{array}{c} 2.306239\\ \begin{array}{c} 2.314882\\ \begin{array}{c} 2.275503\\ \begin{array}{c} 2.320241\\ 2.317203 \end{array} \end{array} \end{array} \end{array}\right],\;$$

(A9)
$$EIM_{S_{2}}=\left[\begin{array}{c} 0.999993\\ \begin{array}{c} 0.997939\\ \begin{array}{c} 0.996792\\ \begin{array}{c} 1.754697\\ 1.537389 \end{array} \end{array} \end{array} \end{array}\right].\;$$

It is seen that the entropy underlying the delay map of *S*_2_ (see Equation (A9)) is much less than that of *S*_1_ (see Equation (A8)). This means that *S*_2_ exhibits a systematic behavior compared to *S*_1_, or *S*_1_ is more random by nature compared to *S*_2_.

## Appendix B. Analyzing Sensor Signals under Different Sampling Windows (Mild Steel (S15CK) Specimen)

Consider the cutting force signal obtained while machining mild steel (S15CK), i.e., *F*_*W*_2__. Figure A2 shows its time series. As seen in Figure A2, the sample size of *F*_*W*_2__ is denoted as *L*_*W*_2__, where *L*_*W*_2__ = 7501. *F*_*W*_2__ is sampled four times using four different sampling windows (light blue colored regions in the time series of *F*_*W*_2__). This results in four new signals, denoted as *F*_*W*_2_*S*_1__, …, *F*_*W*_2_*S*_4__. The corresponding sample sizes are denoted as *L*_*W*_2_*S*_1__, …, *L*_*W*_2_*S*_4__, where *L*_*W*_2_*S*_1__ = 5001, *L*_*W*_2_*S*_2__ = 2501, *L*_*W*_2_*S*_3__ = 1501, and *L*_*W*_2_*S*_4__ = 501.

For the sake of analysis, the signals *F*_*W*_2__ and *F*_*W*_2_*S*_1__, …, *F*_*W*_2_*S*_4__ are transferred to the frequency domain (using FFT) and delay domain (using *d* = 1, as described in Section 3). As such, Figure A3 shows the time series, FFT, and delay map for *F*_*W*_2__. Figure A4, Figure A5, Figure A6 and Figure A7 show the time series, FFT, and delay map for *F*_*W*_2_*S*_1__, …, *F*_*W*_2_*S*_4__, respectively.

As seen in Figure A3b, the prominent frequencies underlying *F*_*W*_2__ are: 0 Hz, 780 Hz, 1560 Hz, 2333.333 Hz, 3113.333 Hz, 3893.333 Hz, and 4673.333 Hz. As seen in Figure A4b, the prominent frequencies underlying *F*_*W*_2_*S*_1__ are: 0 Hz, 780 Hz, 1560 Hz, 2340 Hz, 3110 Hz, 3890 Hz, and 4670 Hz. As seen in Figure A5b, the prominent frequencies underlying *F*_*W*_2_*S*_2__ are: 0 Hz, 780 Hz, 1560 Hz, 2340 Hz, and 3120 Hz. As seen in Figure A6b, the prominent frequencies underlying *F*_*W*_2_*S*_3__ are: 0 Hz, 766.6667 Hz, 1566.6667 Hz, 2333.333 Hz, and 3100 Hz. As seen in Figure A7b, the prominent frequencies underlying *F*_*W*_2_*S*_4__ are: 0 Hz, 800 Hz, 1600 Hz, 2300 Hz, and 3100 Hz. This means that the frequency information varies with the sampling window. In addition, the FFT pattern gets affected when the sampling window is shorter (see Figure A7b compared to Figure A3b).

On the other hand, the delay maps shown in Figure A3c, Figure A4c, Figure A5c, Figure A6c and Figure A7c exhibit similar characteristics under different sampling windows. In particular, the returns of points from one to another are identical. This means that the underlying nature of the *F*_*W*_2__ and *F*_*W*_2_*S*_1__, …, *F*_*W*_2_*S*_4__ are the same, regardless of the sample size. In addition, the density of points underlying the delay maps provides meaningful insight into the sample size of the signal. For example, the density of the delay map shown in Figure A7c is lighter than that of in Figure A3c, which means that the corresponding signal *F*_*W*_2_*S*_4__ (see Figure A7a) undergoes a low sampling window compared to the *F*_*W*_2__ (see Figure A3a).

## Appendix C. Analyzing Sensor Signals under Different Sampling Windows (Ductile Cast Iron (FCD) Specimen)

Consider the cutting force signal obtained while machining ductile cast iron (FCD), i.e., *F*_*W*_3__. Figure A8 shows its time series. As seen in Figure A8, the sample size of *F*_*W*_3__ is denoted as *L*_*W*_3__, where *L*_*W*_3__ = 7501. *F*_*W*_3__ is sampled four times using four different sampling windows (light blue colored regions in the time series of *F*_*W*_3__). This results in four new signals, denoted as *F*_*W*_3_*S*_1__, …, *F*_*W*_3_*S*_4__. The corresponding sample sizes are denoted as *L*_*W*_3_*S*_1__, …, *L*_*W*_3_*S*_4__, where *L*_*W*_3_*S*_1__ = 5001, *L*_*W*_3_*S*_2__ = 2501, *L*_*W*_3_*S*_3__ = 1501, and *L*_*W*_3_*S*_4__ = 501.

For the sake of analysis, the signals *F*_*W*_3__ and *F*_*W*_3_*S*_1__, …, *F*_*W*_3_*S*_4__ are transferred to the frequency domain (using FFT) and delay domain (using *d* = 1, as described in Section 3). As such, Figure A9 shows the time series, FFT, and delay map for *F*_*W*_3__. Figure A10, Figure A11, Figure A12 and Figure A13 show the time series, FFT and delay map for *F*_*W*_3_*S*_1__, …, *F*_*W*_3_*S*_4__, respectively.

As seen in Figure A9b, the prominent frequencies underlying *F*_*W*_3__ are: 0 Hz, 780 Hz, 1560 Hz, 2333.333 Hz, 3113.333 Hz, 3893.333 Hz, and 4673.333 Hz. As seen in Figure A10b, the prominent frequencies underlying *F*_*W*_3_*S*_1__ are: 0 Hz, 780 Hz, 1560 Hz, 2340 Hz, 3110 Hz, 3890 Hz, and 4670 Hz. As seen in Figure A11b, the prominent frequencies underlying *F*_*W*_3_*S*_2__ are: 0 Hz, 780 Hz, 1560 Hz, 2340 Hz, and 3120 Hz. As seen in Figure A12b, the prominent frequencies underlying *F*_*W*_3_*S*_3__ are: 0 Hz, 766.6667 Hz, 1566.6667 Hz, 2333.333 Hz, and 3100 Hz. As seen in Figure A13b, the prominent frequencies underlying *F*_*W*_3_*S*_4__ are: 0 Hz, 800 Hz, 1600 Hz, 2400 Hz, and 3100 Hz. This means that the frequency information varies with the sampling window. In addition, the FFT pattern gets affected when the sampling window is shorter (see Figure A13b compared to Figure A9b).

On the other hand, the delay maps shown in Figure A9c, Figure A10c, Figure A11c, Figure A12c and Figure A13c exhibit similar characteristics under different sampling windows. In particular, the returns of points from one to another are identical. This means that the underlying nature of the *F*_*W*_3__ and *F*_*W*_3_*S*_1__, …, *F*_*W*_3_*S*_4__ are the same, regardless of the sample size. In addition, the density of points underlying the delay maps provides meaningful insight into the sample size of the signal. For example, the density of the delay map shown in Figure A13c is lighter than that of Figure A9c, which means that the corresponding signal *F*_*W*_3_*S*_4__ (see Figure A13a) undergoes a low sampling window compared to the *F*_*W*_3__ (see Figure A9a).

## Appendix D. Analyzing Sensor Signals under Different Time Latencies (Mild Steel (S15CK) Specimen)

Consider the cutting force signal obtained while machining mild steel (S15CK), i.e., *F*_*W*_2__(*t*), *t* = 0, Δ*t*, 2Δ*t*, …, *m* × Δ*t* (can also be seen in Figure 6). As mentioned before, here Δ*t* is the sampling period of 0.02 ms. To incorporate time latency or delay, Δ*t* is increased using the delay parameter *d* (non-zero integer), such as *d* × Δ*t*. For example, for *d* = 1, the sampling period remains 1 × 0.02 = 0.02 ms; for *d* = 2, the sampling period becomes 2 × 0.02 = 0.04 ms; and alike. As mentioned in Section 3, *d* × Δ*t* is simplified using *D*, where *D* = *d* × Δ*t*. As such, a set of time series is generated using *D* where *d* = 1, 2, …. The goal is to understand the dynamics underlying the cutting force. For this, the time series datasets are transferred to the frequency domains (using FFT) and corresponding delay domains. Table A1 shows the outcomes for some of the delays, i.e., *d* = 1, 5, 10, 20, 30, 40, 50, and 60.

As seen in Table A1, when *d* increases, the frequency information underlying the *F*_*W*_2__ gets affected. The prominent frequencies (see the FFT diagram for *d* = 1) are gradually lost due to aliasing [30] when *d* > 5 (see the FFT diagrams for *d* = 10, 20, 30, 40, 50, and 60).

On the other hand, consider the delay maps consisting of the points (*F*_*W*_2__(*t*),*F*_*W*_2__(*t*+*D*)) shown in Table A1. When *d* = 1, the delay map exhibits a very systematic pattern. When *d* increases, the delay maps get more and more scattered (see the delay maps for *d* = 5, 10, 20, 30, 40, and 50). This means that the signal gets more and more chaotic due to the presence of delay. However, when *d* = 60, the corresponding delay map is somewhat systematic and similar to that of *d* = 5. This means that the underlying natures of these two signals are similar, regardless of the difference in the sampling rate. It is worth mentioning that the corresponding FFT plots (see the FFT plots for *d* = 5 and for *d* = 60 shown in Table A1) are different and do not preserve the nature of the source signal. As such, delay domain-based representation is more informative for understanding the underlying nature of *F*_*W*_2__ under a low data acquisition rate due to time latency.

**Table A1:** *F*_*W*_2__ in the form of time series, FFT, and delay map under varying delay (*d*).

Time Series	FFT	Delay Map
*d* = 1		

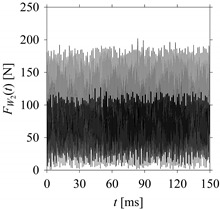
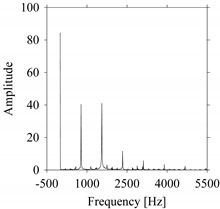
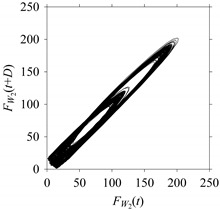
		
*d* = 5		

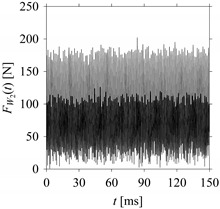
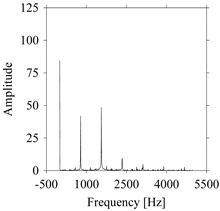
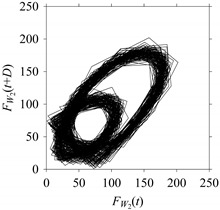
		
*d* = 10		

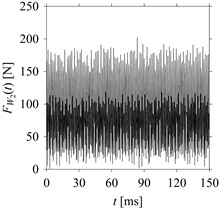
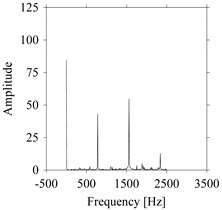
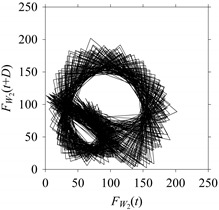
		
*d* = 20		

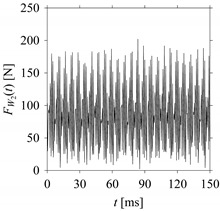
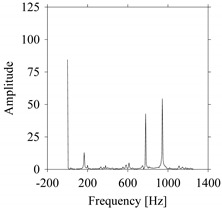
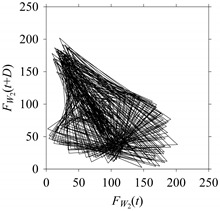
		
*d* = 30		

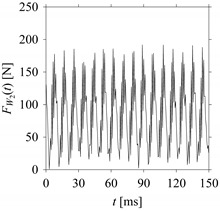
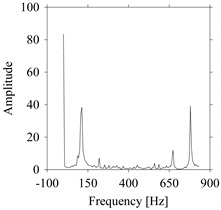
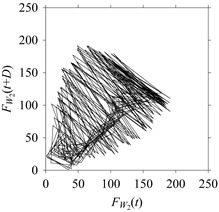
		
*d* = 40		

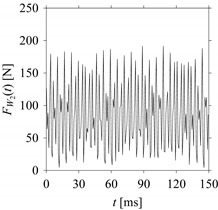
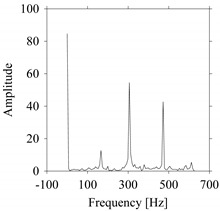
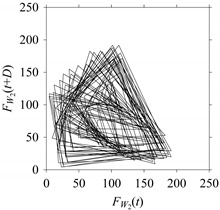
		
*d* = 50		

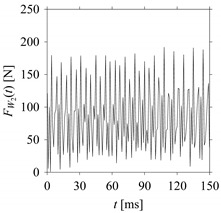
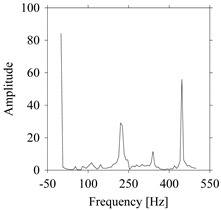
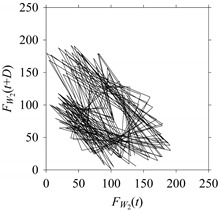
		
*d* = 60		

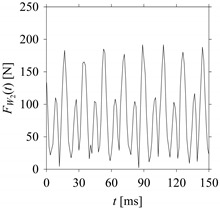
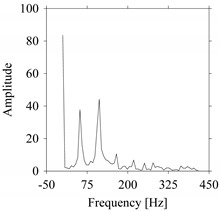
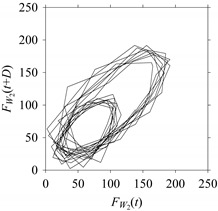
		

## Appendix E. Analyzing Sensor Signals under Different Time Latencies (Ductile Cast Iron (FCD) Specimen)

Consider the cutting force signal obtained while machining ductile cast iron (FCD), i.e., *F*_*W*_3__(*t*), *t* = 0, Δ*t*, 2Δ*t*, …, *m* × Δ*t* (can also be seen in Figure 6). As mentioned before, here Δ*t* is the sampling period of 0.02 ms. To incorporate time latency or delay, Δ*t* is increased using the delay parameter *d* (non-zero integer), such as *d* × Δ*t*. For example, for *d* = 1, the sampling period remains 1 × 0.02 = 0.02 ms; for *d* = 2, the sampling period becomes 2 × 0.02 = 0.04 ms; and alike. As mentioned in Section 3, *d* × Δ*t* is simplified using *D*, where *D* = *d* × Δ*t*. As such, a set of time series is generated using *D* where *d* = 1, 2, …. The goal is to understand the dynamics underlying the cutting force. For this, the time series datasets are transferred to the frequency domains (using FFT) and the corresponding delay domains. Table A2 shows the outcomes for some of the delays, i.e., *d* = 1, 5, 10, 20, 30, 40, 50, and 60.

As seen in Table A2, when *d* increases, the frequency information underlying the *F*_*W*_3__ gets affected. The prominent frequencies (see the FFT diagram for *d* = 1) are gradually lost due to aliasing [30] when *d* > 5 (see the FFT diagrams for *d* = 10, 20, 30, 40, 50, and 60).

On the other hand, consider the delay maps consisting of the points (*F*_*W*_3__(*t*),*F*_*W*_3__(*t*+*D*)) shown in Table A2. When *d* = 1, the delay map exhibits a very systematic pattern. When *d* increases, the delay maps get more and more scattered (see the delay maps for *d* = 5, 10, 20, 30, 40, and 50). This means that the signal gets more and more chaotic due to the presence of the delay. However, when *d* = 60, the corresponding delay map is somewhat systematic and similar to that of *d* = 5. This means that the underlying natures of these two signals are similar, regardless of the difference in the sampling rate. It is worth mentioning that the corresponding FFT plots (see the FFT plots for *d* = 5 and *d* = 60 shown in Table A2) are different and do not preserve the nature of the source signal. As such, delay domain-based representation is more informative for understanding the underlying nature of *F*_*W*_3__ under a low data acquisition rate due to time latency.

**Table A2:** *F*_*W*_3__ in the form of time series, FFT, and delay map under varying delay (*d*).

Time Series	FFT	Delay Map
*d* = 1		

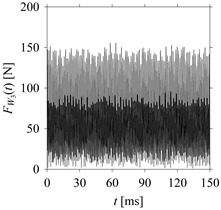
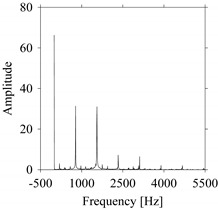
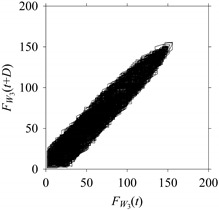
		
*d* = 5		

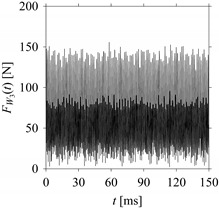
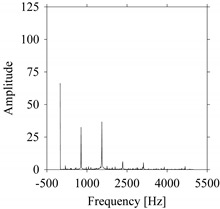
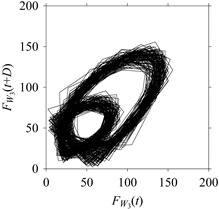
		
*d* = 10		

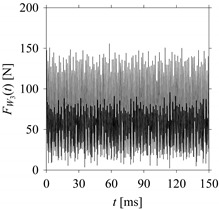
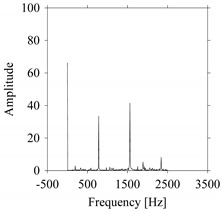
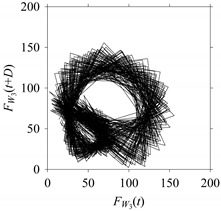
		
*d* = 20		

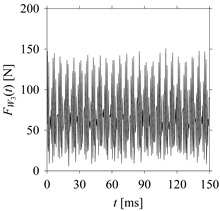
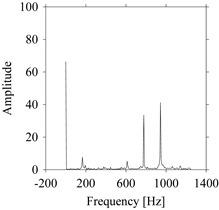
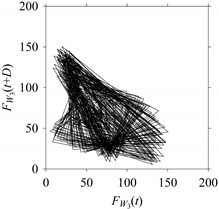
		
*d* = 30		

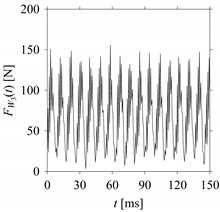
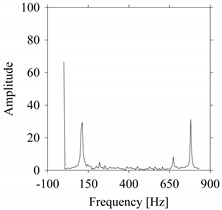
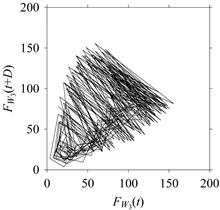
		
*d* = 40		

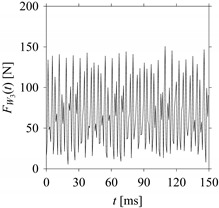
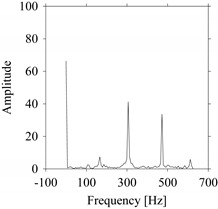
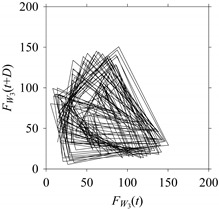
		
*d* = 50		

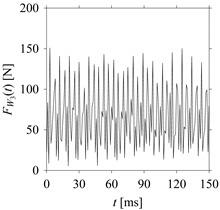
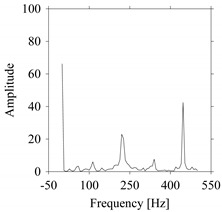
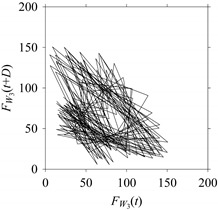
		
*d* = 60		
		
